# Host cellular factors involved in pseudorabies virus attachment and entry: a mini review

**DOI:** 10.3389/fvets.2023.1314624

**Published:** 2023-11-27

**Authors:** Lei Tan, Kaixin Wang, Ping Bai, Shuo Zhang, Mengting Zuo, Xianghua Shu, Aibing Wang, Jun Yao

**Affiliations:** ^1^College of Animal Science, Yangtze University, Jingzhou, China; ^2^College of Veterinary Medicine, Hunan Agricultural University, Changsha, China; ^3^Yunnan Southwest Agriculture and Animal Husbandry Group Co., Ltd, Kunming, China; ^4^Hunan Provincial Key Laboratory of the TCM Agricultural Biogenomics, Changsha Medical University, Changsha, Hunan, China; ^5^College of Animal Medicine, Yunnan Agricultural University, Kunming, China; ^6^Yunnan Tropical and Subtropical Animal Virus Diseases Laboratory, Yunnan Animal Science and Veterinary Institute, Kunming, China

**Keywords:** pseudorabies virus, cellular factors, involvement, viral attachment and entry, antiviral strategies

## Abstract

Pseudorabies virus (PRV) belongs to the *Alphaherpesvirinae* subfamily and serves as an exceptional animal model for investigating the infection mechanism of Herpes simplex virus type 1. Notably, PRV has the capability to infect a wide range of mammals, including humans, highlighting its potential as an overlooked zoonotic pathogen. The attachment and entry steps of PRV into host cells are crucial to accomplish its life cycle, which involve numerous cellular factors. In this mini review, we offer a comprehensive summary of current researches pertaining to the role of cellular factors in PRV attachment and entry stages, with the overarching goal of advancing the development of novel antiviral agents against this pathogen.

## Introduction

Pseudorabies virus (PRV), belonging to the subfamily *Alphaherpesvirinae*, is an enveloped double-stranded DNA virus (1). A variety of mammals, such as pigs, wild boars, goats, cattle, dogs, cats, and minks, are susceptible to the infection of PRV (2). Only pigs and wild boars are the unique nature hosts for PRV, clinical symptoms of pseudorabies (PR) caused by PRV in pigs are primarily characterized by central neural disorders in piglets with high morbidity, reproductive diseases in pregnant sows (2). Moreover, the prevalence of PRV also poses a huge threat to humans, with numerous of human encephalitis or endophthalmitis cases caused by PRV infection recently documented in China (3). Unfortunately, effective antiviral agents for treating PRV infections in both humans and animals remain limited.

Similar to other viruses, PRV infection involves multiple steps, including viral attachment, entry, replication, assembly, extracellular trafficking, and viral egress (4). Among these processes, viral attachment and entry are the initial steps in completing the virus’s life cycle. Importantly, the virus could interact with or hijack various cellular factors to facilitate its attachment and entry efficiency. Thus, understanding the involvement of these cellular factors or their interactions with viral proteins during virus attachment and entry is critical for developing novel strategies to combat this pathogen. Numerous cellular proteins/factors have been reported to play roles in PRV attachment and entry stages, including Human HveC (Nectin-1) (5), Nectin-2 (6), Neuropilin-1 (NRP1) (7), Niemann-Pick1 (NPC1) (8, 9), porcine paired immunoglobulin-like type 2 receptor α (PILRα) (10) and beta (PILRβ) (11), etc. Meanwhile, a variety of cellular factors have been identified to inhibit viral attachment and entry, including the cholesterol 25-hydroxycholesterol (CH25H) (12), IFN-induced transmembrane protein 1 (IFITM1) (13), and IFITM2 (14).

**Figure 1:**
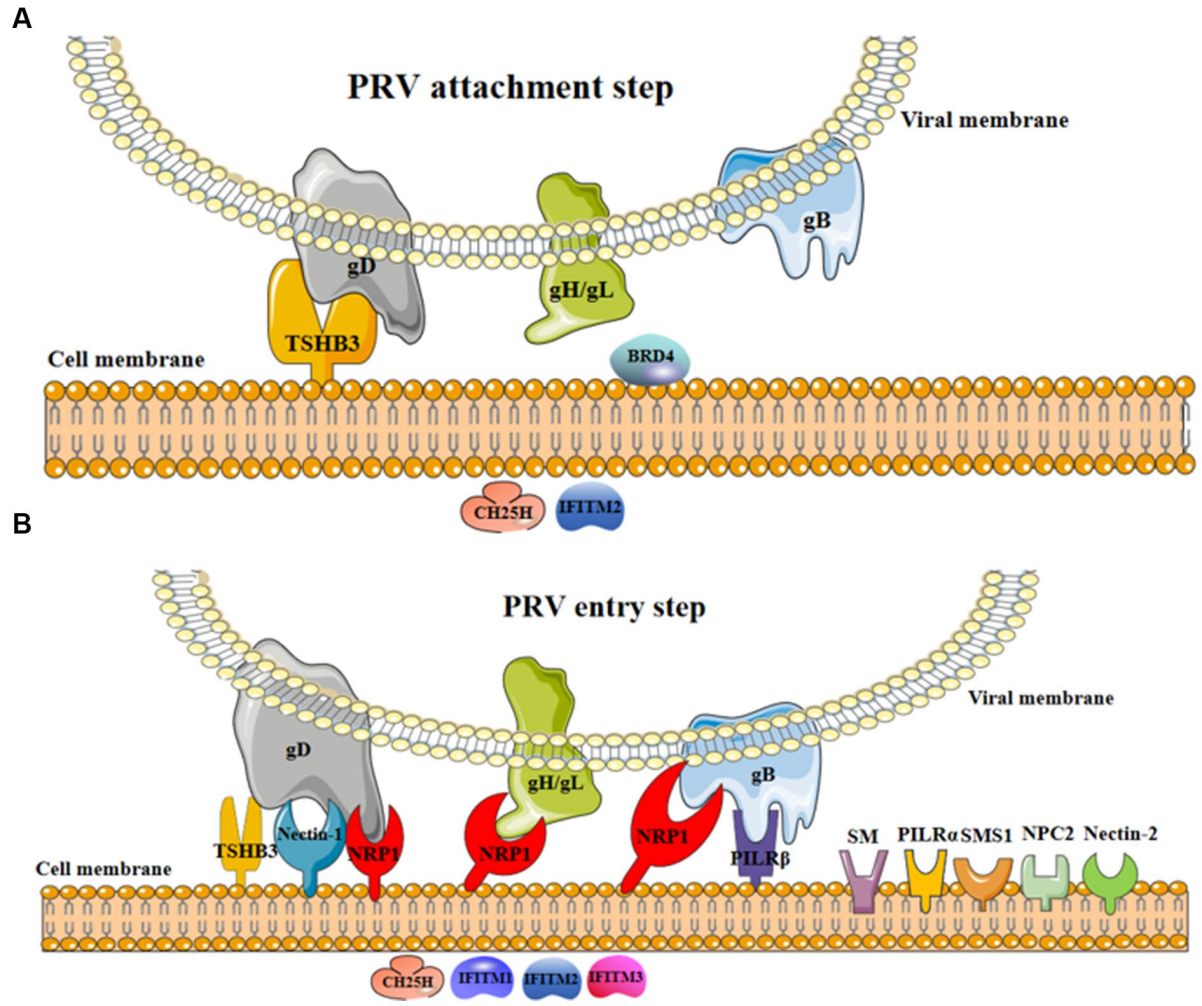
The involvement of cellular factors in PRV attachment and entry steps. **(A)** In the process of viral attachment, the interaction of cellular TSHB3 and PRV gD promoted viral attachment (17); BRD4 might promote viral attachment (21); CH25H and IFITM2 were restricted factors limiting viral attachment (12, 14). **(B)** In the process of viral entry, the interaction of cellular TSHB3 (gD) (17), nectin-1 (gD) (5), NRP1 (gD, gH/gL, and gB) (7), and PILRβ (gB) (11) and PRV glycoproteins promote viral entry into host cells; nectin-2 (6), NPC1 (5, 8), PILRα (18), SM (19), and SMS1 (20) were potential factors promoting viral entry; CH25H (12) and IFITM1-3 (13, 14) were restricted factors inhibiting viral entry.

In this mini review, we provide a comprehensive summary of the latest information focusing on cellular factors involved in PRV attachment and entry stages (Table 1; Figure 1). This summary aims to offer new insights for developing novel strategies against PRV infection, such as antiviral agents.

**Table 1:** Function and antiviral strategies against cellular factors involved in PRV attachment and entry.

Factor	Function	Antiviral strategies
Nectin-1	Entry receptor Nectin-1 directly interacted with PRV gD (5)	Gene-modified mice (15, 16)
Nectin-2	Entry receptor? Knockout of nectin-2 suppressed PRV infection *in vitro* (6)	NA
NRP1	Attachment and entry receptor NRP1 directly interacted with the gB, gD, and gH (7)	NA
NPC1	Entry receptor? NPC1 inhibitor treatment blocked PRV entry *in vitro* and showed anti-PRV activity *in vivo* (mice) (5, 8)	Inhibitor (5, 8)
THBS3	Attachment and entry co-receptor THBS3 directly interacted with the PRV gD (17)	Antibody and soluble protein (17)
PILRα	Entry receptor? PILRα antibody blocked PRV entry (18)	Antibody (18)
PILRβ	Attachment or entry receptor? PRV gB directly interacted with PILRβ to mediate NK cells cytotoxicity (11)	NA
SM	Entry receptor? SM inhibitor suppressed PRV entry *in vitro* (19)	Inhibitor (19)
SMS1	Entry receptor? Knockout of SMS1 inhibited PRV entry *in vitro* (20)	NA

## Cellular factors facilitating PRV attachment and entry

Viral attachment and entry steps are pivotal in establishing the virus life cycle within host cells, and they also partly determine the specificity of tissue or host cell infection (22, 23). These processes involve interactions between viral glycoproteins (e.g., gB, gC, gD, gH/gL, etc.) and cellular factors on the host cell membrane, facilitating the viral absorption and entry into host cells (23).

### Nectin1 and nectin-2

Nectin-1 or nectin-2 are members of the nectin family, characterized by three Ig-like domains in the ectodomain (IgV-IgC-IgC), as well as transmembrane and cytoplasmic regions (24). These proteins are widely expressed in all tissues of mammals and involved in cell–cell adhesion (24). Growing evidence supports their roles as primary receptors for various *Alphaherpesvirus* infection *in vitro* and *in vivo*. For instance, Krummenacher et al. revealed that the C-terminal region of HSV-1 gD interacted with the N-terminal region of nectin-1, facilitating HSV-1 entry into host cells (25). Soluble nectin-1 protein treatment inhibited HSV-1 entry into different cell lines (5). Deletion of nectin-1 in animal experiments prevented viral infection and significantly alleviated clinical symptoms caused by HSV-1 or HSV-2 infection (26). Nectin-2 plays similar roles in HSV-1 infection compared to nectin-1 (27).

Both nectin-1 and nectin-2 are essential cellular factors for PRV infection. CHO-K1 cells, which lack *Alpherpesvirus* receptors, are resistant to PRV infection. Li et al. found that over-expression of nectin-1 in CHO-K1 cells promoted PRV entry (5). Further investigation revealed that PRV gD directly interacted with both human and swine nectin-1, with higher binding affinity observed for human nectin-1 (5). Considering the high amino acid homology (96%) between porcine and human nectin-1, it is plausible that human nectin-1 may participate in PRV cross-transmission from pig to humans (5).

Another study generated nectin-1 or nectin-2 knockout (KO) PK15 cells via CRISPR/Cas9 technology, and found that these KO cells exhibited greater resistance to PRV infection compared with wild-type cells (6). Interestingly, further research showed that the deletion of nectin-1 or nectin-2 reduced the cell-to-cell spread ability of PRV, without affecting viral absorption and entry steps (6).

Moreover, nectin-1 mutant (F129A) mice presented milder clinical symptoms, decreased viral loads in tissue samples, and lower mortality rates when infected with PRV (16). Additionally, transgenic mice expressing soluble form of porcine nectin-1 protein were resistant to PRV infection (15). Consequently, nectin-1 represents an ideal target for combating PRV both *in vitro* and *in vivo*, through developing antibodies and chemical inhibitors against nectin-1, even generating nectin-1 gene-modified pigs, which may offer novel approaches against PRV infection in the future.

### Neuropilin-1

NRP1 is a cell-surface receptor involved in a variety of biological processes, including angiogenesis, regulating vascular permeability, nervous system development, and tumorigenesis. NRP1 also acts as an essential co-receptor promoting the entry and replication stages of various viruses, such as Kaposi’s sarcoma-associated herpesvirus (KSHV) (21), SARS-CoV-2 (28), Epstein–Barr virus (EBV) (29). However, a recent research showed that NRP1 was a restricting factor inhibiting HIV attachment of progeny virions to target cells (30).

The involvement of NRP1 in PRV infection has been elucidated recently. Chen et al. first demonstrated that over-expression of NRP1 increased the production progeny viruses in PRV-infected cells, while inhibiting the endogenous expression of NRP1 suppressed viral replication in SK-N-SH cells (7). Further analysis revealed that over-expression of NRP1 enhanced viral attachment and entry efficiency into CHO cells, indicating that NRP1 might promote PRV entry (7). Furthermore, a cell-to-cell fusion assay revealed that NRP1 over-expression promoted viral glycoprotein-mediated cell-to-cell fusion (7). Co-immunoprecipitation (Co-IP) and BiFC assays indicated that NRP1 directly interacted with the gB, gD, and gH, suggesting that NRP1 promoted PRV attachment and entry by interacting with these viral glycoproteins (7). Moreover, PRV gB was found to accelerate NRP1 degradation via a lysosome-dependent pathway and this process was dependent on its furin-cleavage activity (7). Collectively, these findings underscore the essential roles of NRP1 in PRV attachment and entry into host cells, and suggest that NRP1 inhibitors could be effective agents for PRV prevention and treatment.

### Niemann-pick C1

NPC1 belongs to the cholesterol family that is essential for the lysosomal cholesterol transport from late endosomes to cellular membrane (31). Abnormal expression of NPC1 is associated with various cancers (32, 33). Recently, the contribution of NPC1 to virus infection has garnered attention, and the NPC1-specific inhibitor, U18666A, has been widely used to explore the potential roles of NPC1 in viral infection (34).

Li et al. first investigated the antiviral activities of inhibitors targeting proteins involved in lipid metabolism against PRV infection and found that U18666A inhibited PRV proliferation *in vitro* (8). Furthermore, viral replication ability was significantly suppressed in NPC1-knockout PK15 cells, while this effect was reversed by the over-expression of wild-type NPC1 in NPC1-knockout cells (8). However, no significant difference in PRV proliferation was observed between wide-type and NPC1-knockout cells after U18661A treatment, indicating that U18666A inhibited PRV infection via a NPC1-dependent pathway (8). Further investigation revealed that U18666A treatment primarily blocked viral entry by decreasing cholesterol aggregation in the plasma membrane, thus inhibiting the biological activities of clathrin-coated pits (8). Importantly, U18666A treatment improved the survival rates of PRV-infected mice by decreasing cytokines production and viral loads in different tissues (8). Overall, these results suggested that NPC1 is involved in PRV entry. However, another study suggested that U18666A treatment suppressed PRV infection by inhibiting the release of PRV particles (9). Thus, cellular NCP1 might participate in multiple stages of PRV life cycle, warranting further investigation.

### Thrombospondin 3

Thrombospondin 3 (THBS3) is a member of the THBS family involved in cell–cell and cell-matrix interactions, and participating in the development of skeletal muscle. Additionally, the knockout of THBS3 in mice increases the stability and production of integrin membranes, providing protection against disease-causing stimuli for the heart (35).

Pan et al. first identified THBS3 as a novel co-receptor for PRV entry into cells (17). Following a strategy similar to the exploration of NPC1’s role in PRV infection, Pan et al. investigated the effects of THBS3 knockdown, knockout, and over-expression on PRV proliferation. The results revealed that siRNA targeting THBS3 or THBS3 knockout effectively inhibited PRV-GFP (a recombinant PRV strain expressing GFP) infection in different cell lines (17). Moreover, both THBS3 antibody and soluble THBS3 protein treatment demonstrated similar antiviral activities against PRV-GFP infection, while THBS3 over-expression promoted PRV-GFP infection in PK15 cells (17).

Co-IP and pull-down assays demonstrated that both the N and C terminals of THBS3 directly interacted with PRV gD, but not gC and gB (17). And THBS3 over-expression promoted PRV binding/attachment to PK15 and CHO cells, with no impact on the expression and cellular location of nectin-1 (17). However, over-expression of THBS3 enhanced nectin-1 mediated viral fusion and entry efficiency (17). Considering the direct interaction between gD and THBS3 during PRV infection, and the multiple roles of THBS3 in viral infection, the potential of THBS3 as an antiviral target *in vivo* needs further exploration in the future.

### Porcine paired immunoglobulin-like 2 receptor alpha and beta (PILRα and PILRβ)

Porcine paired immunoglobulin-like 2 receptors (PILRs) belong to the member of the immunoglobulin superfamily, consist of two subtypes, PILRα and PILRβ. The genetic sequences of PILRα and PILRβ are conserved among different mammal species, yet their regulatory activities in the innate and adaptive immune systems differ. PILRα and PILRβ are widely expressed in various immune system-related cells, including the dendritic cells, NK cells, monocytes, etc. Importantly, these receptors have drawn significant attention due to their involvements in *Alphaherpesvirus* infection.

Satoh et al. found that CHO-K1 cells with PILRα over-expression were effectively infected with HSV-1 and PRV, while the infection abilities of HSV-1 and PRV were completely inhibited after PILRα antibody treatment (10, 18). Further investigation revealed that PILRα participated in HSV-1 infection by interacting with gB during viral entry step (10).

Concerning PILRβ, Pelsmaeker et al. found that expression of PRV gB accelerated the NK cell-mediated killing of gB-transfected swine kidney cells, which was also observed in PRV-infected cells (11). Further flow cytometric analysis demonstrated that PRV gB increased the binding activity of recombinant PILRβ protein to the gB-transfected cells (11). These results underscore the essential roles of PILRβ in PRV infection-mediated NK cell toxicity.

### Sphingomyelin

Sphingomyelin (SM) is a primary component of the phospholipids found in the mammalian plasma membrane, actively contributing to the formation of lipid rafts in conjunction with the cholesterol (36). Pastenkos et al. made the noteworthy discovery that treatment with *Staphylococcus aureus*-derived sphingomyelinase (SMase) resulted in robust inhibition of PRV entry, as SMase treatment significantly reduced SM staining intensity, signifying the crucial role of SM in PRV entry (19). Furthermore, a recent study demonstrated that the knockout of sphingomyelin synthase 1 (SMS1) led to a significant inhibition of PRV entry into the rabbit PK13 cells (20).

## Cellular factors inhibiting PRV attachment and entry

During viral entry, PRV glycoproteins (such as gB and gH) mediate membrane fusion processes that facilitate the penetration of viral capsid into the cytoplasm (37). Subsequently, the viral DNA genome is transported to the host nucleus, where it replicates, triggering host antiviral immune responses, as thoroughly summarized in recent reviews (38, 39). Some cellular factors involved in the innate immune response exhibit antiviral activities during PRV attachment or/and entry step.

### IFN-induced transmembrane proteins

The IFITMs family, comprising five subtypes (IFITM1, IFITM2, IFITM3, IFITM5, and IFITM10) in humans, is conserved and mainly localized in the endo-lysosomal and plasma membranes. IFITMs are involved in various processes, including stem cell properties, DNA damage, and the activation of innate immune processes (40). Swine IFITMs family (IFITM1, IFITM2, and IFITM3) have been shown to inhibit multiple virus infections, including PRV (13, 14).

Wang et al. demonstrated that IFITM1 transcription was significantly up-regulated in PRV-infected cells (PK15 and 3D4/21 cells) (13). Knockdown of IFITM1, but not IFITM2 and IFITM3, enhanced PRV replication in PK15 cells, while over-expression of IFITM1 displayed antiviral activity (13). Further analysis revealed that IFITM1 knockdown promoted PRV entry into the target cells, suggesting that IFITM1 acts as a restricting factor limiting PRV entry, although its impact on PRV attachment requires further investigation (13).

Another study indicated that PRV infection significantly up-regulated the transcription of IFITM1, IFITM2, and IFITM3 at 12 h post-infection (hpi) and 24 hpi (14). Over-expression of IFITM1, IFITM2, or IFITM3 inhibited PRV replication, while knockdown of these IFITMs enhanced PRV replication efficiency (14). Further research demonstrated that all three IFITM subtypes restricted PRV entry into cells, with IFITM2 specifically interfering with PRV binding efficiency, a process that depends on cholesterol accumulation (14).

### Cholesterol 25-hydroxylase

CH25H is a multi-transmembrane endoplasmic reticulum-associated enzyme responsible for catalyzing cholesterol into 25-hydrocholesterol (25HC) (41). CH25H belongs to the interferon-stimulated genes and broadly resists viral infection via different pathways (42).

Wang et al. reported that PRV infection increased the CH25H mRNA levels at 12 hpi and 24 hpi. Subsequent research showed that CH25H overexpression or 25HC treatment suppressed PRV replication (12). Further investigations, utilizing TCID_50_ and western blot assays, revealed that 25HC treatment suppressed PRV attachment and entry steps (12). Collectively, the results suggest that CH25H negatively affected PRV replication by interfering with viral attachment and entry (12).

### Bromodomain protein 4

Bromodomain protein 4 (BRD4), a member of the bromodomain and Extra-Terminal domain (BET) family, possesses a bromodomain that can bind to acetylated histones, participating in various cellular processes such as DNA repair, replication, and transcription (21). Moreover, the functions of BRD4 on PRV infection have received attention recently (21).

Wang et al. initially found that BRD4 inhibitors exhibited anti-PRV infection ability through GFP-reporter assays. Inhibition of BRD4 did not affect the transcription of viral genes but significantly suppressed PRV attachment (21). BRD4 inhibitor treatment or knockdown significantly inhibited PRV attachment, as revealed by RT-qPCR and western blot assays (21). Moreover, pre-treatment of JQ-1, a BRD4 inhibitor, increased the survival rate of PRV-infected mice compared to the control group (21). Mechanistically, BRD4 inhibitor treatment induced chromatin decompaction and double DNA damage, subsequently activating cGAS-dependent innate immune responses (21).

## Perspective and concluding remarks

As of now, PRV continues to be a significant pathogen, causing substantial financial losses in the global swine industry. Furthermore, the potential for PRV transmission from pigs to other animal species has raised concerns, even prompting public alarm regarding the virus’s potential risk to humans. Like other *Alphaherpesvirueses*, PRV can establish latency in swine, thus making it challenging to eradicate through vaccination efforts.

Intracellular oblige pathogens, including viruses, depend on cellular components to accomplish their life cycles (8). Among the critical stages for viral infection, attachment and entry represent ideal targets for the development of antiviral strategies, akin to generating CD163 gene knockout pigs for PRRSV control (43). In the case of PRV, nectin-1 has been extensively studied as a cellular receptor for PRV entry and/or cell-to-cell spread, and genetic modification targeting nectin-1 holds promise for antiviral activities against PRV in mouse models. Thus, it is reasonable to expect that nectin-1 gene-edited pigs would be resistant to PRV infection, although ongoing monitoring of the clinical performance of these gene-edited pigs is essential, given the multiple roles of nectin-1. Moreover, the development of antibodies and inhibitors against nectin-1 could be effective approach for PRV treatment, since the antiviral activity of antibodies against PRV or HSV-1 has been observed *in vitro* (39, 44).

Apart from nectin-1 and nectin-2, various cellular proteins involved in the promotion of PRV attachment and entry have been identified, partly due to the extensive research into the infection mechanisms of other *herpesviruses* and public concerns about PRV. However, it remains unclear which PRV-encoded proteins are involved in binding or interacting with the newly identified cellular factors, such as NPC1, SM, and SMS1 (8, 19, 20). Addressing these concerns will deepen our understanding of viral pathogenesis, and facilitate the development of vaccines and antiviral agents.

Additionally, this review has summarized four cellular factors negatively regulating PRV attachment and entry steps, including interferon-stimulated genes (IFITM1, IFITM2, and CH25H) and BRD4. However, several questions require clarification: (1) Further investigations should be performed to confirm the antiviral activities of these cellular factors against PRV *in vivo.* (2) It has been reported that PRV tegument proteins and glycoproteins can suppress the innate immune responses induced by virus infection (45). Such as PRV UL24, which can directly inhibit the transcription of multiple interferon-stimulated genes (e.g., OASL and ISG20 genes) (46). Therefore, further research is needed to determine whether PRV-encoded proteins can directly interact with or reduce the expression level of these cellular proteins (IFITM1, IFITM2, CH25H, and BRD4).

Functionally, cellular factors played similar roles in *Herpesvirus* infection, such as NRP1, which was identified as an entry factor promoting different *Herpesvirus* infection, including PRV (7), EBV (29), and KSHV (21). While NRP1 was recently identified as an antiviral agent inhibiting HIV infection, mainly via suppressing the infectivity of HIV-1 progeny virions and the viral transmission ability (30). NRP1 inhibitors effectively suppressed PRV infection *in vitro*, however, the co-infection of PRV and other pathogens were frequently detected in clinical samples (47). Further efforts will explore the roles of PRV attachment or entry-related cellular factors in other swine virus’ infection, to comprehensively assess the possibility of these cellular factors in developing antiviral agents.

## Conclusion

In summary, the prevalence of PRV remains a global concern, posing significant risks to human health. Recent researches have shed light on the roles of cellular factors in PRV attachment and entry steps, providing valuable insights for the development of novel antiviral approaches. However, our current understanding of PRV attachment and entry mechanisms is still incomplete. Therefore, further efforts are required to identify additional cellular factors involved in PRV attachment and entry, and explore their effects. Moreover, there is an urgent need to develop innovative antiviral agents such as chemical inhibitors, antibodies, and peptides, that can effectively target cellular factors like nectin-1 and nectin-2, which play crucial role in PRV attachment and entry. These advancements will undoubtedly contribute to the prevention and control of PRV in the future.

## Author contributions

LT: Writing – original draft, Writing – review & editing. KW: Data curation, Writing – review & editing. PB: Manuscript revision & diagram preparation. SZ: Manuscript preparation, Review & Modification-polish. MZ: Writing – review & editing. XS: Funding acquisition, Writing – review & editing. AW: Funding acquisition, Writing – review & editing. JY: Funding acquisition, Writing – review & editing.
